# Supplementary material for: Profiling of G-Protein Coupled Receptors in Adipose Tissue and Differentiating Adipocytes Offers a Translational Resource for Obesity/Metabolic Research
Source: Cells. 2023 Jan 19;12(3):377. doi: 10.3390/cells12030377 (PMC9913134; doi:10.3390/cells12030377)
Supplement: Supplementary file 1 [file cells-12-00377-s001.zip › cells-2067582-supplementary.pdf]

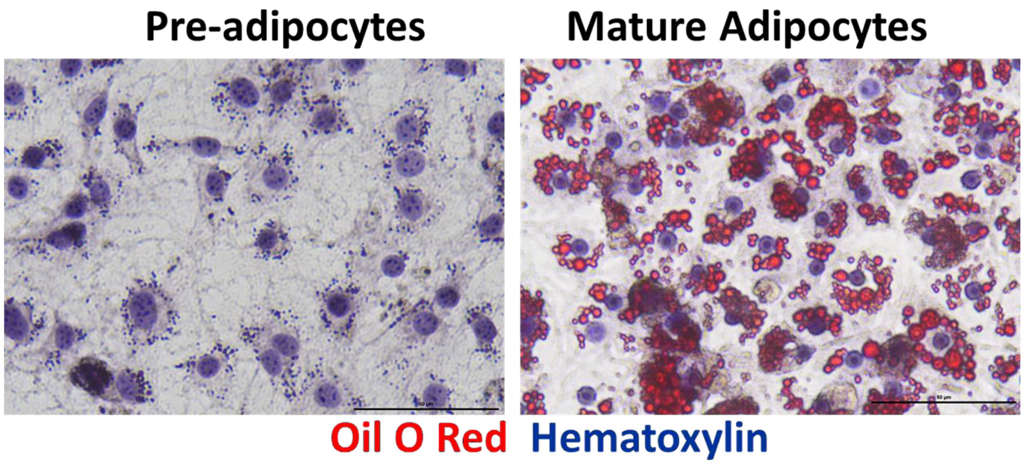

Figure S1. Oil-O red staining of 3T3-L1 cells before and after differentiation.

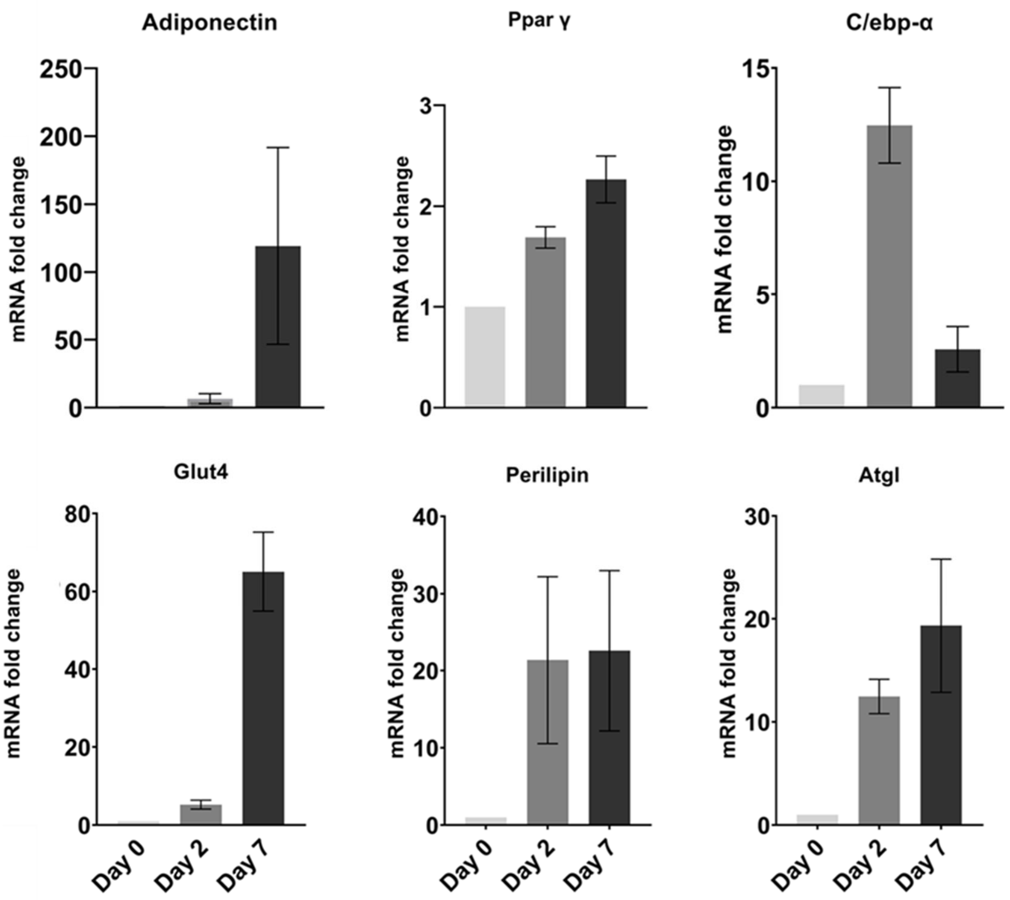

Figure S2. Adipogenic differentiation of 3T3 L1 cells and expression of key adipogenic markers. mRNA expression C/EBP-α and PPAR-γ, Glut4, Hsl, Atgl, Adiponectin receptor and Adiponectin during the differentiation of 3T3 L1 adipocytes. Each bar represents an average of three independent experiments.

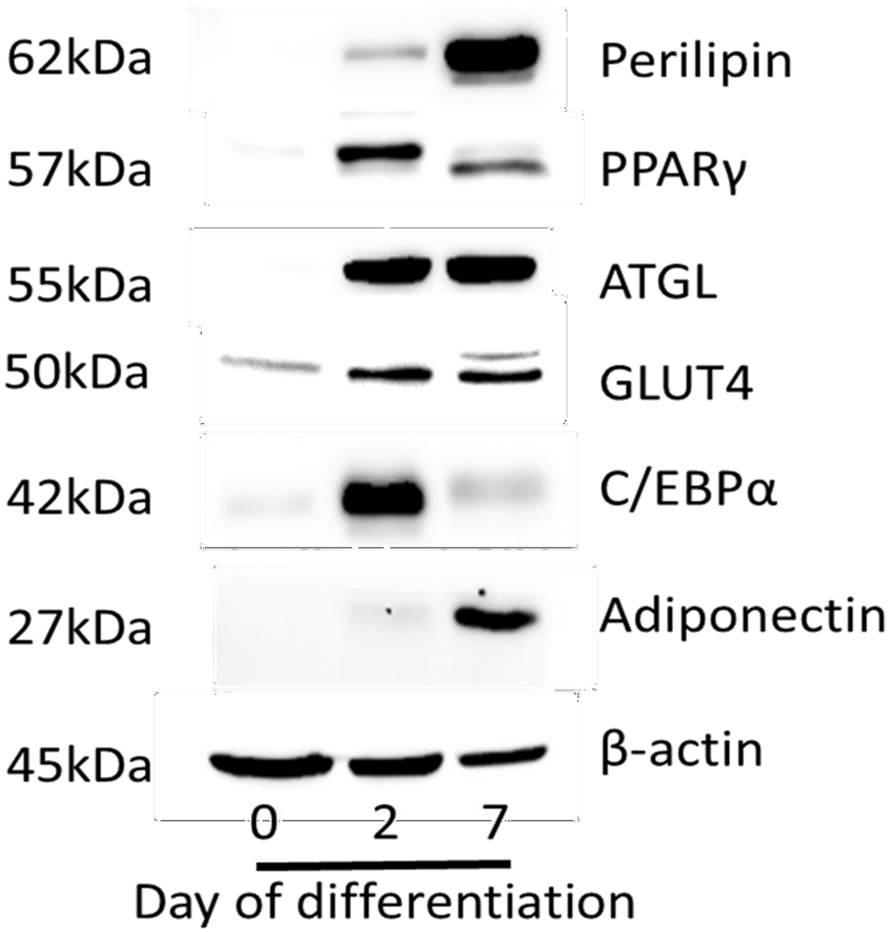

Figure S3. Adipogenic differentiation of 3T3 L1 cells and expression of key adipogenic markers. Protein expression C/EBP-α and PPAR-γ, Glut4, Hsl, Atgl, Adiponectin receptor and Adiponectin during the differentiation of 3T3 L1 adipocytes.

Table S1. Comparison between changes in the expression of GPCRs in human obesity (Dataset # GSE 152992) and Diet-induced obesity in mice (Dataset # 142187).

Upregulated in Both	Downregulated in Both	Divergent Expression Changes
ACKR2	ADCYAP1R1	CALCRL
ADGRE1	ADGRB3	DRD1
ADGRE2	ADRB3	AVPR1A
ADGRE5	HCAR1	FPR1
ADGRG4	HCAR2	
ADORA3	EDNRA	
C3AR1		
C5AR1		
CCR2		
CCR3		
CCR5		
CCR7		
CELSR1		
CXCR3		
CXCR4		
F2R		
F2RL2		
FZD1		
FZD2		
GPR132		
GPR141		
GPR146		
GPR183		
GPR84		
GPR85		
GPR88		
LPAR5		
P2RY10		
PTAFR		
PTGER4		
P2RY14		
MRGPRE		
